# Clinical and genetic analysis of methylmalonic aciduria in 60 patients from Southern China: a single center retrospective study

**DOI:** 10.1186/s13023-024-03210-0

**Published:** 2024-05-15

**Authors:** Ling Su, Huiying Sheng, Xiuzhen Li, Yanna Cai, Huifen Mei, Jing Cheng, Duan Li, Zhikun Lu, Yunting Lin, Xiaodan Chen, Minzhi Peng, Yonglan Huang, Wen Zhang, Li Liu

**Affiliations:** Department of Genetics and Endocrinology, Guangzhou Women and Children’s Medical Center, Guangzhou Medical University, Tianhe District, Guangzhou, Guangdong 510623 P.R. China

**Keywords:** Methylmalonic aciduria, MMACHC gene, MMUT gene, SUCLA2 gene, Pulmonary hypertension

## Abstract

**Background:**

Methylmalonic aciduria (MMA) is a group of rare genetic metabolic disorders resulting from defects in methylmalonyl coenzyme A mutase (MCM) or intracellular cobalamin (cbl) metabolism. MMA patients show diverse clinical and genetic features across different subtypes and populations.

**Methods:**

We retrospectively recruited 60 MMA patients from a single center and diagnosed them based on their clinical manifestations and biochemical assays. We then performed genetic analysis to confirm the diagnosis and identify the causal variants.

**Results:**

We confirmed the common clinical manifestations of MMA reported previously. We also described four rare MMA cases with unusual symptoms or genetic variants, such as pulmonary hypertension or limb weakness in late-onset patients. We identified 15 MMACHC and 26 MMUT variants in 57 patients, including 6 novel MMUT variants. Two patients had only one MMAA variant each, and one patient had mild MMA due to mitochondrial DNA depletion syndrome caused by a SUCLA2 variant. Among 12 critically ill patients, isolated MMA was associated with higher C3, blood ammonia, and acidosis, while combined MMA was linked to hydrocephalus on skull MRI. MMACHC c.658-660delAAG and MMUT c.1280G > A variants were correlated with more severe phenotypes.

**Conclusions:**

Our study demonstrates the clinical and genotypic heterogeneity of MMA patients and indicates that metabolic screening and genetic analysis are useful tools to identify rare cases.

## Introduction

Methylmalonic aciduria (MMA) is a rare group of genetic metabolic disorders caused by defects in methylmalonyl coenzyme A mutase (MCM) or intracellular cobalamin (cbl) metabolism. These defects lead to the accumulation of methylmalonic acid and methylcitrate in the body, which cause heterogeneous clinical manifestations affecting multiple organs [1]. MMA can be classified into two main types: combined MMA and isolated MMA, depending on the presence or absence of homocysteinemia. The incidence of MMA ranges from 1/20000–1/25000 worldwide [2], but varies among regions in China, from nearly 1/5589 to 1/35734 [3].

Combined MMA is characterized by high levels of methylmalonic acid and homocysteinemia, and is caused by impaired synthesis of both adenosylcobalamin and methylcobalamin. This particular form of MMA may be caused by variants in several genes, including MMACHC, MMADHC, LMBRD1, ABCD4 and HCFC1. These genes correspond to different subtypes: cblC, cblD, cblF, cblJ and cblX, respectively [4, 5]. The most prevalent subtype of combined MMA is cblC, which arises from variants in the MMACHC gene. This subtype accounts for 70% of all MMA patients in China [5]. Clinical presentations of combined MMA can vary significantly, ranging from fetal onset in utero to atypical onset in adults. However, most patients exhibit symptoms before the age of one, including anemia, growth retardation, epilepsy and metabolic dysfunction [3, 6].

Isolated MMA is caused by deficiency of MCM or its coenzyme component due to variants in the genes MMUT, MMAA, MMAB and MMADHC [4, 7]. Variants in the genes SUCLG1 and SUCLA2 cause mitochondrial DNA depletion syndrome, which may be associated with mild isolated MMA, a distinct type of isolated MMA [8, 9]. The main feature of isolated MMA is high levels of methylmalonic acid without homocysteinemia [10]. The main gene affected in isolated MMA is MMUT. The clinical presentation of isolated MMA is similar to that of combined MMA, but the treatment and prognosis differ between the two types, and are also influenced by the genotype of the patients [3].

In this study, we conducted a retrospective analysis of the clinical and genetic features of MMA patients in our center from 2014 to August 2023. We also reported uncommon cases and rare types of patients with variants in the MMAA and SUCLA2 genes. We focused on the clinical presentation, biochemical results, and genotype of critically ill children with onset. Our aim was to improve the diagnosis and management of MMA patients and to elucidate the molecular mechanisms underlying this complex disorder.

## Methods

### Patients

We retrospectively enrolled 60 MMA patients (38 with MMA with homocysteinemia and 22 with isolated MMA) who were diagnosed in Guangzhou Women and Children’s medical center from 2014 to August 2023. They mainly came from Guangdong province. The diagnosis was based on clinical presentation and biochemical assays, and confirmed by genetic analysis.

We collected general data on the age at onset and diagnosis, clinical manifestations, treatment and outcomes. Among them, 12 critically ill children received treatment in NICU and PICU at onset. We compared the clinical phenotypes and genotypes of 38 patients with MMA with homocysteinemia and 22 patients with isolated MMA, and explored the association between genotype and severity of onset.

This study was approved by the Ethics Committee of Guangzhou Women and Children’s medical center. Written informed consents were obtained from the parents of study participants.

### Biochemical analysis

All patients underwent routine bloodtests, blood gas-electrolyte analysis, blood ammonia, cardiac-hepatic-renal function tests. Some early patients with hematological involvement underwent bone marrow puncture to confirm the etiology. Urinary organic acids, including methylmalonic acid and methylcitric acid were measured by gas chromatography-mass spectrometry. Blood levels of acylcarnitines, including propionylcarnitine (C3) and acetylcarnitine (C2) and amino acids were detected by MS/MS. The concentration of plasma tHcy was also determined by chemiluminescence method. Almost all of these biochemical analyses were performed at the time of diagnosis.

### Genetic analysis

Peripheral blood samples were collected from the patients who were suspected of having classical MMA due to elevated propionylcarnitine in serum and elevated methylmalonic acid levels and methylcitric acids in the urine. DNA was extracted using a reagent kit according to the protocol. The exons and exon-introns of MMACHC and MMUT genes were amplified and detected by Sanger sequencing. Next-generation sequencing was also used for mutations screening in small patient cohorts. The pathogenicity assessment of novel variants followed the American College of Medical Genetics and Genomics (ACMG) standards and guidelines. Q-PCR was used to verify patients who were suspected of large deletions.

### Imaging examination

Most patients underwent cranial CT or cranial MR examination. Patients with convulsions underwent electroencephalogram examination. Some patients underwent brainstem auditory and visual evoked potential examination. Critically ill children hospitalized in PICU and NICU underwent chest X-ray examination and chest CT examination.

### Treatment

During the acute decompensation period, we considered methylmalonic acidemia (MMA) with homocysteinemia when a high amount of urinary methylmalonic acid and methylcitric acid levels were detected, and C3 and C3/C2 ratios, along with plasma total homocysteine (tHcy), were elevated. In such cases, patients should promptly receive adequate treatment with hydroxycobalamin (1-10 mg per day) and betaine (100–500 mg/kg per day). Additionally, a hydroxycobalamin loading test was recommended. Isolated MMA was considered when only urinary methylmalonic acid and methylcitric acid were detected, and C3 and C3/C2 ratios were elevated, but plasma tHcy remained normal. For these patients, we recommended special formula supplementation and hydroxycobalamin may be administered to assess their responsiveness. In cases of severely elevated blood ammonia, patients temporarily restricted protein intake and received intravenous arginine. If necessary, hemofiltration was employed. Simultaneously, symptomatic supportive treatment included intravenous or oral glucose for caloric supplementation, correction of electrolyte imbalance and acidosis, and intravenous L-carnitine (50–100 mg/kg per dose, twice daily). Critically ill children admitted to the PICU and NICU might require rescue and advanced life support treatment.

During the stable period, patients who respond to hydroxycobalamin should continue intramuscular or subcutaneous injections of hydroxocobalamin, and follow a normal diet. Patients who do not respond to hydroxycobalamin should adhere to strict dietary restriction and L-carnitine treatment. The individual metabolic treatment should be adjusted according to their clinical condition.

## Result

### Clinical features of MMA

#### Clinical characteristics

We identified 22 males and 16 females with combined MMA, who had elevated levels of blood C3, ratio of C3/C2, urine MMA and plasma tHcy. We also identified 10 males and 12 females with isolated MMA, who met the diagnostic criteria of MMA, but had normal or near-normal plasma tHcy. Of the 60 patients, 46 visited the hospital before 12 months of age, and 14 of them developed symptoms before 1 month of age. Six cases with combined MMA and four cases with isolated MMA were asymptomatic and detected by newborn screening (Table 1).

**Table 1:** Overview of MMA patients

	Gene	MMACHC	MMUT	MMAA	SUCLA2
Age of diagnosis	< 1 M	7	7	0	0
	1 M-12 M	23	8	0	1
	> 12 M	8	4	2	0
Gender	M	22	8	1	1
	F	16	11	1	0
Detected in NS		6	4	0	0
Total		38	19	2	1

*MMA* Methylmalonic aciduria, *MMACHC* methylmalonic aciduria and homocystinuria type C protein, *MMUT* Methylmalonyl-CoA mutase, *MMAA* methylmalonic aciduria type A protein, *SUCLA2* solute carrier family 25 member 32, *NS* newborn screening

Patients identified through newborn screening and those diagnosed clinically exhibit significant clinical disparities. Newborn-screened patients are often asymptomatic and rarely encounter metabolic crises during their initial assessments. In contrast, clinically detected patients frequently present symptoms affecting multiple organ systems, particularly during catabolic stress episodes. Among the 50 patients studied, the most prevalent clinical manifestations included psychomotor retardation (24/50), encephalopathy (17/50), feeding difficulties, seizures (16/50), anemia/pancytopenia (12/50), cough/dyspnea (10/50), and vomiting (9/50). In the subgroup of 32 clinically detected patients with combined MMA, common manifestations comprised psychomotor retardation (16/32), feeding difficulties (10/32), and seizures (10/32). Among the 18 patients with isolated MMA, encephalopathy (9/18), psychomotor retardation (8/18), and vomiting (8/18) were most frequent. Additional clinical features included eye problems, recurrent acidosis, and hypoglycemia. Notably, the clinical presentations in this cohort closely align with previously reported findings.

#### Uncommon MMA cases

We present four cases of patients with methylmalonic acidemia (MMA) with different clinical manifestations and genetic variants.

**Fig. 1:**
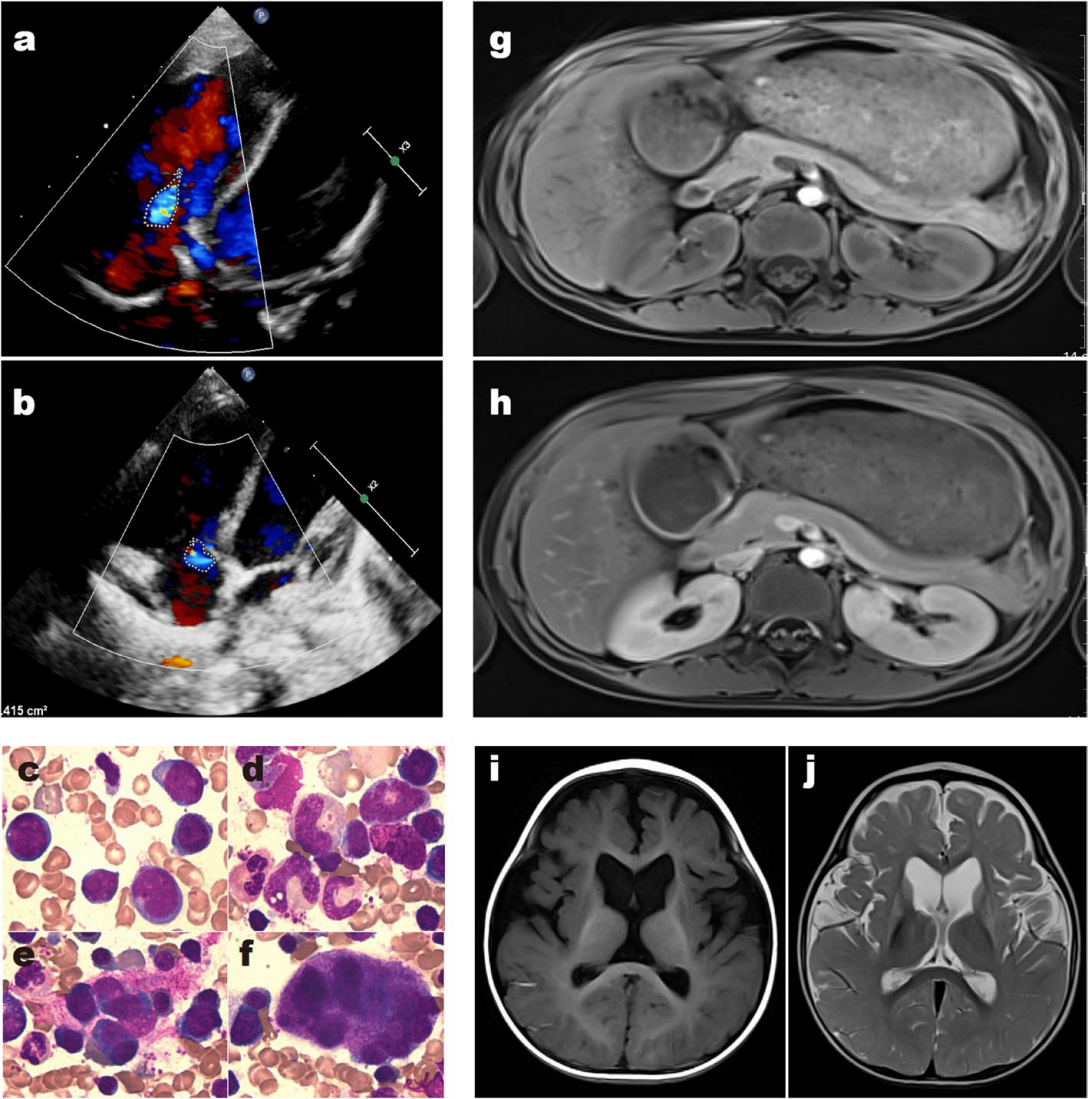
Case 1: Ultrasound suggests moderate to severe pulmonary hypertension, mild tricuspid regurgitation, minimal pericardial effusion (**a**). The image below presents the patient’s echocardiogram taken four months later, indicating a transition to mild levels of both pulmonary arterial hypertension and tricuspid regurgitation (**b**); Case 2: Bone marrow images show moderate anemia and increased bone marrow cells (**c**-**f**). Case 3: The images depict the enhancement of the anterior and posterior roots of the horizontal spinal nerves at levels T12-L2. The ‘**g**’ image represents the transverse weighted imaging (TWI) scan, while the ‘**h**’ image illustrates the enhanced TWI. Case 4: Symmetrical signal abnormalities and atrophy in the bilateral basal ganglia, with dilation of the supratentorial ventricles. Slight enhancement of the meninges in the left frontal region (**i**, **j**)

- Case 1: A two-year-old patient initially presented with wheezing and cyanosis. Doppler echocardiography revealed severe pulmonary hypertension and moderate pericardial effusion, leading to hospital admission. Subsequent metabolic tests showed elevated C3 and plasma tHcy levels, indicating combined methylmalonic acidemia (MMA). Whole exome sequencing confirmed the diagnosis by identifying MMACHC gene variants. The patient received hydroxycobalamin and betaine, which improved his condition and reduced his C3 and plasma tHcy levels. However, he still had cyanosis and required oxygen therapy. We suspected that the cyanosis was due to severe pulmonary hypertension, and we consulted with a multidisciplinary team to prescribe drugs to control it. He was discharged from the hospital without oxygen inhalation and was advised to have regular outpatient visits. The results of his cardiac ultrasound are shown in the Fig. 1. Cardiovascular manifestations, such as arrhythmia and cardiomyopathy, are uncommon in MMA patients. MMA cases with pulmonary hypertension as the initial cardiovascular presentation are even rarer. However, this case highlights the possibility of distinct causes of pulmonary hypertension..
- Case 2: A four-month-old patient presented with unstable head control and was diagnosed with moderate anemia (HGB 63 g/L, normal range: 105–145). This is a case from 2016, when MMA was not as well recognized as it is now, especially among non-genetic disease specialists. Therefore, he was followed up in the hematology department for “moderate anemia”. He underwent bone marrow aspiration, which showed active bone marrow hyperplasia (Fig. 1), and received repeated blood transfusions from 4 to 11 months of age. At 11 months of age, he had unstable sitting posture and obvious psychomotor developmental delay. Metabolic tests confirmed the diagnosis of MMA due to MMACHC gene variants. Skull MRI showed brain atrophy. After the diagnosis, he received hydroxycobalamin and betaine, which improved his anemia symptoms significantly. However, he developed secondary epilepsy at two years of age. Anemia is actually a relatively common clinical manifestation of MMA, but when only anemia is present, clinicians are prone to miss or misdiagnose genetic metabolic diseases. Therefore, clinicians need to inquire about the medical history, perform physical examinations, and track the changes in the patient’s condition in detail.
- Case 3: A five-year-old patient was hospitalized due to limb weakness for one month. One month previously, the child developed weakness in both lower limbs accompanied by abnormal gait. The local hospital diagnosed him with Guillain-Barré syndrome and gave him intravenous immunoglobulin. The weakness in both lower limbs improved, but one month later, the child again had weakness in both lower limbs and was admitted to our hospital for treatment. His MRI showed enhancement of the anterior and posterior roots of the spinal nerves at the T12-L2 level, and the cauda equina nerves below the L3 level, consistent with Guillain-Barré syndrome (Fig. 1). However, urinary organic acids and elevated C3 levels suggested the diagnosis of MMA. He was confirmed to have isolated MMA due to MMUT gene variants complicated by inflammatory demyelinating polyradiculoneuropathy. He received intravenous immunoglobulin and prednisone, which improved his limb movement significantly. He was advised to follow a low protein diet and special formula supplementation because of hydroxycobalamin nonresponsiveness. This child was incidentally found to have MMA due to other comorbidities. The clinical manifestations of late-onset MMA are atypical.
- Case 4: A nine-month-old boy came to the hospital because of unstable head control. He had elevated levels of lactic acid (6.18 mmol/L, normal range < 2 mmol/L) and lactic acid/pyruvate ratio (22.40, normal range 6–14), indicating mitochondrial dysfunction. His skull MRI revealed abnormal symmetric signal and atrophy of the bilateral basal ganglia, suggesting brain damage (Fig. 1). His mitochondrial DNA detection was normal, excluding primary mitochondrial DNA mutations. He was diagnosed with mitochondrial DNA depletion syndrome and mild isolated MMA due to SUCLA2 gene variants after whole exome sequencing. This is a rare condition with only a few reported cases in the literature. The patient received supportive care and nutritional therapy, but his prognosis was poor.

### Mutational spectrum of MMA

All patients underwent genetic testing, and 38 patients were genetically confirmed as having combined MMA, caused by MMACHC gene variants. 19 patients had isolated MMA caused by MMUT gene variants. 2 patients had heterozygous variants in MMAA gene, and 1 patient had mitochondrial DNA depletion syndrome caused by SUCLA2 gene variant, with mild isolated MMA (Table 2).

**Table 2:** Clinical and genetic characteristics of 60 patients with MMA in China in this study

Patient	gender	Age at diagnosis	C3 (umol/L) (0.15–0.80)	C3/C2 (< 0.53)	tHcy (umol/L) (0–15)	gene	Mutation 1	Mutation 2
1	M	11d	7.46	1.26	264.5	MMACHC	c.398_399delAA(p.Gln133Argfs*5)	c.609G > A(p.Trp203*)
2	F	2y	2.866	0.04	81.5	MMACHC	c.609G > A(p.Trp203*)	c.609G > A(p.Trp203*)
3	M	7 m	2.783	0.29	143.3	MMACHC	c.80A > G(.Gln27Arg)	c.394C > T(.Arg132*)
4	M	2y	3.103	0.26	NA	MMACHC	c.609G > A(p.Trp203*)	c.609G > A(p.Trp203*)
5	M	1 m	6.64	1.52	NA	MMACHC	c.398_399delAA(p.Gln133Argfs*5)	c.609G > A(p.Trp203*)
6	F	1m23d	12.694	3.98	330	MMACHC	c.567dupT(p.Ile190Tyrfs*13)	c.567dupT(p.Ile190Tyrfs*13)
7	M	13d	13.76	1.04	221	MMACHC	c.481C > T(.Arg161*)	c.609G > A(p.Trp203*)
8	F	1 m	14.54	1.4	185.5	MMACHC	c.567dupT (p.Ile190Tyrfs*13)	c.609G > A(p.Trp203*)
9	M	4 m	28.16	1.39	93.2	MMACHC	c.80A > G(.Gln27Arg)	c.609G > A(p.Trp203*)
10	F	1 m	10.69	1.59	189	MMACHC	c.217C > T(p.Arg73*)	c.658_660delAAG(p.Lys220del)
11	M	2 m	7.83	1.99	271	MMACHC	c.567dupT(p.Ile190Tyrfs*13)	c.609G > A(p.Trp203*)
12	M	23d	15.66	2.46	NA	MMACHC	c.658-660delAAG(p.Lys220del)	c.658-660delAAG(p.Lys220del)
13	M	1m29d	17.58	1.33	149.2	MMACHC	c.609G > A(p.Trp203*)	c.609G > A(p.Trp203*)
14	F	1 m	8.34	0.66	220	MMACHC	c.609G > A(p.Trp203*)	c.609G > A(p.Trp203*)
15	F	3 m	13.49	2.52	187.5	MMACHC	c.609G > A(p.Trp203*)	c.609G > A(p.Trp203*)
16	M	21d	10.66	2.05	130	MMACHC	c.398_399delAA(p.Gln133Argfs*5)	c.567dupT(p. Ile190Tyrfs*13)
17	F	17d	7.645	0.61	196.5	MMACHC	c.609G > A(p.Trp203*)	c.658-660delAAG(p.Lys220del)
18	F	1y1m	14.73	3.64	109.8	MMACHC	c.609G > A(p.Trp203*)	c.609G > A(p.Trp203*)
19	M	2 m	6.19	0.44	105	MMACHC	c.609G > A(p.Trp203*)	c.609G > A(p.Trp203*)
20	F	6 m	NA	NA	NA	MMACHC	c.80A > G(p.Gln27Arg)	c.609G > A(p.Trp203*)
21	M	4 m	6.94	0.33	163	MMACHC	c.609G > A(p.Trp203*)	large heterozygous deletion
22	F	9 m	8.1	2.49	108	MMACHC	c.328_331delAACC(p.Asn110Aspfs*13)	c.609G > A(p.Trp203*)
23	F	4 m	9.47	2.89	210	MMACHC	c.609G > A(p.Trp203*)	c.609G > A(p.Trp203*)
24	M	4y	13.46	7.49	104	MMACHC	c.394C > T (p.Arg132*)	c.658-660delAAG(p.Lys220del)
25	M	1y	9.09	3.31	98	MMACHC	c.609G > A(p.Trp203*)	c.658-660delAAG(p.Lys220del)
26	F	6 m	21	0.08	118	MMACHC	c.658_660delAAG(p.Lys220del)	c.658_660delAAG(p.Lys220del)
27	F	3 m	8.45	0.86	156	MMACHC	c.315C > G (p.Tyr105*)	c.616C > T (p.Arg206Trp)
28	F	15d	10.96	1.52	86	MMACHC	c.467G > A (p.Gly156Asp)	c.609G > A(p.Trp203*)
29	M	1 m	16.18	0.24	210.98	MMACHC	c.609G > A (p.Trp203*)	c.609G > A (p.Trp203*)
30	M	2 m	28.82	1.24	235	MMACHC	c.398_399delAA(p.Gln133Argfs*5)	c.658_660delAAG(p.Lys220del)
31	F	6y	1.220	0.1	65.8	MMACHC	c.609G > A (p.Trp203*)	c.658-660delAAG(p.Lys220del)
32	M	2y9m	5.3	0.46	NA	MMACHC	c.80A > G(p. Gln27Arg)	c.609G > A(p.Trp203*)
33	M	1 m	6.83	0.07	73.6	MMACHC	c.398_399delAA(p.Gln133Argfs*5)	c.626dupT(p.Thr210Aspfs*35)
34	F	2 m	17.39	2.28	146	MMACHC	c.609G > A(p.Trp203*)	c.609G > A(p.Trp203*)
35	M	2 m	5.34	1.09	113	MMACHC	c.609G > A(p.Trp203*)	c.658_660delAAG(p.Lys220del)
36	M	15d	16.2	3.74	203	MMACHC	c.80A > G(p. Gln27Arg)	c.567dupT (p.Ile190Tyrfs*13)
37	M	5y	8.26	0.6	156.8	MMACHC	c.365A > T(p.His122Leu)	c.609G > A (p.Trp203*)
38	M	2y	12.21	0.88	142.08	MMACHC	c.80A > G (p.Gln27Arg)	c.217C > T (p.Arg73*)
39	F	3y	55.24	3.32	7.5	MMAA	c.730G > T(p.Val244Phe)	NA
40	M	1y1m	2.42	0.77	23.9	MMAA	c.593_596delCTAG(p.Thr198Serfs*6)	NA
41	M	9 m	3.54	0.12	6.62	SUCLA2	c.851G > A (p.Arg284His)	c.851G > A (p.Arg284His)
42	M	3d	48.85	1.89	NA	MMUT	c.1280G > A(p.Gly427Asp)	c.1677-1G > A
43	F	1 m	26.59	6.5	10.5	MMUT	c.1046-1058del13(p.Ala349Aspfs*20)	c.1159A > C(p.Thr387Pro)
44	F	15d	16.75	8.38	5.3	MMUT	c.323G > A(p.Arg108His)	c.544dupA(p.Met182Asnfs)
45	F	2 m	104.75	4.34	2.9	MMUT	c.682C > T(p.Arg228*)	c.1531C > T(p.Arg511*)
46	F	1 m	188.45	3.54	8.8	MMUT	c.1159A > C(p.Thr387Pro)	c.1280G > A(p.Gly427Asp)
47	F	3d	34.17	1.7	7.3	MMUT	c.729_730insTT(p.Asp244Leufs*39)	c.2179C > T(p.Arg727*)
48	M	1 m	37.16	2.21	1.7	MMUT	c.1159A > C(p.Thr387Pro)	c.1159A > C(p.Thr387Pro)
49	F	3 m	33.21	2.32	4.9	MMUT	c.1677-1G > A	c.2179C > T(p.Arg727*)
50	M	7 m	49.25	3.5	6	MMUT	c.323G > A(p.Arg108His)	c.1106G > A(p.Arg369His)
51	F	2 m	21.48	1.82	4.4	MMUT	c.1850 T > G (p.Leu617Arg)	c.1996G > A(p.Val666Met)
52	M	3y	16.43	1.84	4.3	MMUT	c.1280G > A(p.Gly427Asp)	c.1956 + 1G > T
52	M	5d	73.2	2.75	5.5	MMUT	c.1159A > C(p.Thr387Pro)	c.1280G > A(p.Gly427Asp)
54	F	8 m	35.95	5.72	9.2	MMUT	c.1036_1038delCTT(p.Leu346del)	c.1159A > C(p.Thr387Pro)
55	M	5d	4.9	6.7	NA	MMUT	c.1835delG(p.Gly612Valfs*8)	c.925 T > G ( p.Trp309Gly)
56	F	2y4m	8.03	0.51	3.85	MMUT	c.944dupT(p.Tyr316Leufs*11)	c.1084-10A > G HET
57	M	1d	9.13	5.87	2.9	MMUT	c.1294del(p.Glu432Asnfs*12)	c.1888G > A(p.Gly630Arg)
58	F	5y	5.16	0.4	3.75	MMUT	c.2179C > T (p.Arg727*)	c.2251 T > A(p.*751Lys)
59	M	6y	15.21	1.73	8.4	MMUT	c.788G > T(Gly263Val)	c.1097A > G (p.Asn366Ser)
60	F	1 m	15.02	0.81	3.7	MMUT	c.835 T > G(p.Tyr279Asp)	c.1663G > A (p.Ala555Thr)

MMA: MMAA: MMACHC: MMUT: HET

**Fig. 2:**
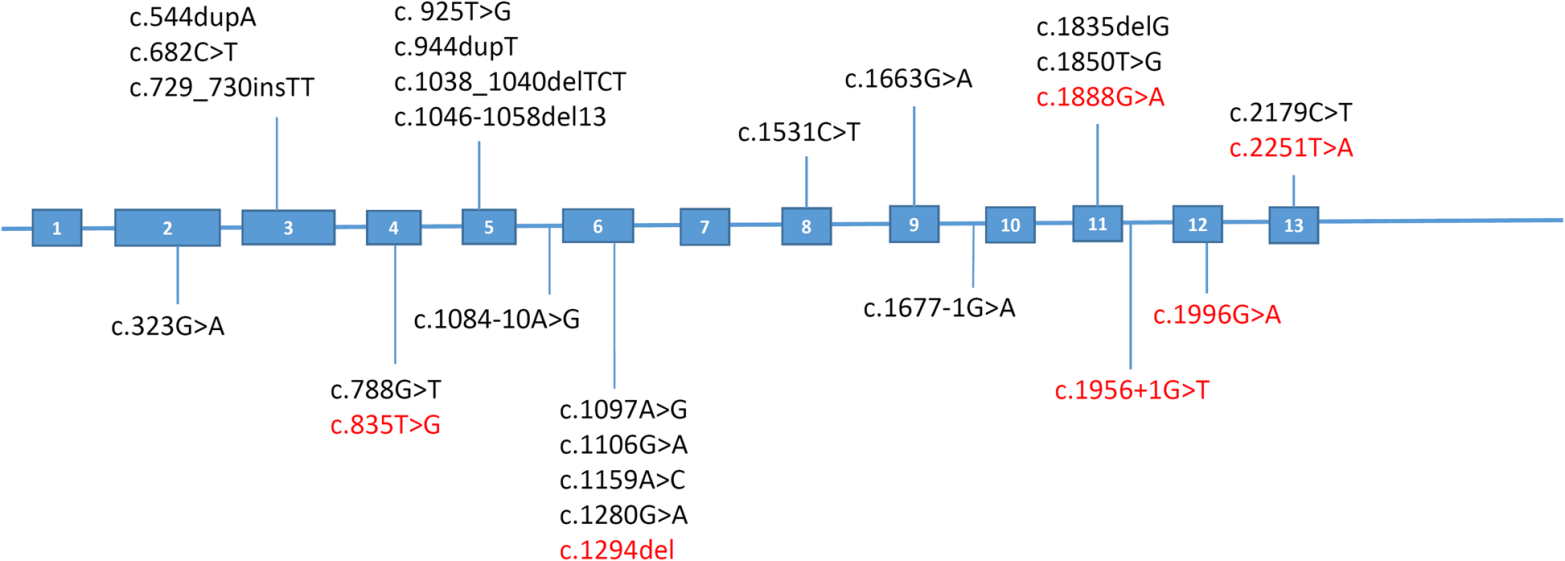
This figure provides a schematic representation of the variant distribution on the MMUT gene identified in our study. It specifically illustrates the locations of the 26 distinct variants we discovered. Variants that are novel to this study are emphasized in red, while the known pathogenic variants are depicted in black

We found a total of 15 MMACHC variants, including 5 nonsense variants, 5 frameshift variants, 4 missense variants, and 1 gene deletion. The common variants were c.609G > A (36/76), c.658_660delAA (11/76), c.567dupT (6/76), c.80A > G 676), c.398_399delAA (5/76), c.394C > T (2/76), c.217C > T (2/76) and others only appeared once. Variant c.609G > A was detected in 10 patients in homozygous form, c.658_660delAA was detected in 2 patients in homozygous form, c.567dupT was detected in 1 patient in homozygous form, and other 12 variants only appeared in heterozygous form. Among the MMUT variants detected in 19 patients with isolated MMA, we found a total of 26 variants, involving 12 missense variants, 8 frameshift variants, 3 nonsense variants and 3 splicing site variants. The common variants were c.1159A > C (6/38), c.1280G > A (4/38), c.2179C > T (3/38), c.1677-1G > A (2/28), c.323G > A (2/28), and others only appeared once. Only one patient with isolated MMA had c.1159A > C homozygous variant, and other patients had compound heterozygous variants. Variants c.835 T > G, c.1888G > A, c.1996G > A, c.1956 + 1G > T, c.1294del, c.2251 T > A were novel and considered likely pathogenic according to ACMG criteria (Fig. 2).

We also analyzed the hotspot variants of MMACHC gene and MMUT gene in other regions of the world (Table 3). The most common variants of the MMACHC gene in the southern region were in line with the general mutation pattern of the Chinese population. However, the most frequent variants of the MMUT gene differed from those in other regions due to ethnic and sample size factors. The incidence of MMA appeared to be lower in the south than in the north of China.

**Table 3:** Reported hotspot mutations of MMACHC gene and MMUT gene in MMA patients from different regions of the world

Gene	Area (case number)	Hot Mutation 1 (proportion)	Hot Mutation 2 (proportion)	Hot Mutation 3 (proportion)	References
MMACHC	Tianjin (44)	c.609G > A (34.09%)	c.658_660delAAG (13.64%)	c.80A > G (13.64%)	Wang et al. 2021 [11]
	Zhenzhou (158)	c.609G > A (48.28%)	c.658_660delAAG (13.17%)	c.567dupT (7.84%)	Hu et al. 2022 [5]
	Canada (118)	c.271dupA (42%)	c.394C > T (20%)	c.331C > T (5%)	Lerner-Ellis et al. 2009 [12]
	Portugal and Italy (41)	c.271dupA (55%)	c.394C > T (16%)	c.331C > T (9%)	Nogueira et al. 2008 [13]
	Beijing (79)	c.609G > A (48.1%)	c.658_660delAAG (13.9%)	c.482G > A (7%)	Liu et al. 2010 [14]
	Shanghai and so on (510)	c.609G > A (41.3%)	c.658_660delAAG (9.2%)	c.482G > A (9.0%)	Ling et al. 2022 [15]
	Guangzhou (35)	c.609G > A (50%)	c.658_660delAA (15.7%)	c.398_399delAA/c.567dupT (7.1%)	This study
MMUT	Beijing and so on (204)	c.729_730insTT (14.2%)	c.1106G > A (7.6%)	c.914 T > C (5.4%)	Yu et al. 2021 [3]
	Switzerland (151)	p.Asn219Tyr(c.655A > T) (7.9%)	p.Arg369His (c.1106G > A) (7.6%)	p.Arg694Trp (c.2080C > T) (3.6%)	Forny et al. 2016 [10]
	Tianjin (8)	c.914 T > C (18.7%)	c.1677-1G > C (18.7%)		Wang et al. 2021 [11]
	Zhenzhou70	c.729_730insTT (16.78%)	c.1106G > A (p.Arg369His) (7.69%)	c.914 T > C (6.99%)	Hu et al. 2022 [5]
	Shanghai (43)	c.729_730insTT (15.4%)	c.1280G > A (NA)	c.1630_1631delGGinsTA (NA)	Han et al. 2015 [16]
	Japan (46)	c.385 + 5G > A (NA)	c.1481 T > A(p.L494X) (NA)	c.349G > A(p.E117*) (NA)	Skamoto et al. 2007 [17]
	Canada (Hispanic) (27)	p.R108C (NA)	p.G94R (NA)	c.1022dupA(NA)	Worgan et al. 2006 [18]
	Canada (black) (29)	p.G717V(NA)	p.G623R (1867G4A) (NA)	p.G94V(NA)	Worgan et al. 2006 [18]
	Guangzhou (14)	c.1159A > C (21.4%)	c.1280G > A (14.3%)	c.1677-1G > A (7.1%)	This study

### Clinical presentation and genotype of critically ill patients

We further analyzed the clinical features and mutation types of 7 severe patients with combined MMA and 5 severe patients with isolated MMA, who were admitted to NICU/PICU at disease onset (Table 4). All patients had early onset before 1 year of age. We defined severe patients as those who had one or more of the following criteria: coma, respiratory failure, shock, and these symptoms were usually accompanied by acidosis, pancytopenia or cerebral edema.

**Table 4:** Clinical and genetic data of 12 critically ill children with MMA

ID	Age of onset	Hospitalization age	Gender	Birth condition	PH	HCO3(mmol/L)	ALT (U/L)	AST (U/L)	Hcy (umol/L)	WBC (10^9^/L)	HGB(g/L)	PLT (109/L)	C3(umol/L)	AMM(umol/L)	Presentation
P42	2d	3d	M	2.86 kg	6.93	3	29	119	NA	10	155	239	45.85	877	Hypoglycemia, poor appetite, poor responsiveness, moaning, severe dyspnea, pale and gray skin, and fatigue
P52	2d	3d	M	2.9 kg	7.34	18.3	30	82	5.5	3.4	95	31	174.228	607	Poor responsiveness, poor feeding, limb tremors on the second day of life, admitted to the neonatal unit of this hospital, blood ammonia level of 171umol/l, diagnosed with hyperammonemia
P50	7 m	7 m	M	3.2 kg	7.11	6.1	16	36	6	8.7	121	310	49.25	140	Coughing for more than 10 days, worsened by dyspnea, vomiting, and poor responsiveness for 3 days, followed by shock; recurrent seizures during observation, characterized by limb stiffness, head shaking, clenched fists, and cyanosis of the lips
P46	1 m	1 m	F	3.2 kg	7.18	6.2	74	314	8.8	0.4	72	6	188.45	577.9	Poor responsiveness for 5 days, fever and dyspnea for 4 days
P45	2 m	2 m	F	2.45 kg	7.39	18	78	67	2.9	2	74	80	104.75	141–327	Vomiting for 5 days, poor responsiveness for more than one day, worsened responsiveness after being hospitalized for 3 days, very weak cry, irregular breathing at about 50 breaths per minute, poor general condition of the child, unstable vital signs; transferred to PICU
P27	3 m	3 m	F	3.1 kg,	7.19	10.1	12	49	156–23	5.96	42	174	8.45	85	Poor responsiveness for 8 days, poor spirit, weak sucking, weak crying, nodding breathing; accompanied by regurgitation and occasional coughing of 1–2 sounds; hospitalized locally; moaning on the second day of hospitalization with upward gaze of both eyes, limb stiffness, occasional limb shaking; unresponsive to calling; fever; transferred to PICU
P12	18d	23d	M	2.65 kg	7.35	20.5	9	15	NA	3.8	102–64	26	15.66	52	Decreased white blood cell and platelet count for 6 days; progressive feeding difficulty; somnolence; decreased activity
P5	1 m	1 m	M	2.65 kg	7.37	34.7	9	31	NA	3.04	76	43	6.64	52	Refusal of milk for 6 days; poor mental responsiveness for 5 days
P19	1m20d	2 m	M	3.1 kg	7.29	20.7	12	15	105	11.9	88	449	6.19	58	Coughing for 11 days; dyspnea; poor mental responsiveness and appetite; abnormal respiratory rhythm on the 10th day of hospitalization; severe dyspnea on the 19th day of hospitalization; cyanosis and tachycardia under high-flow oxygen therapy; transferred to PICU
P21	5 m	5 m	F	2.65	7.274	18	35	33	118	10.6	89	194	21	44	Intermittent coughing and fever for more than half a month; multiple episodes of pneumonia after birth; delayed development; unstable head lifting
P10	1 m	1 m	F	NA	7.38	21.4	18	17	189	4.2	58	504	10.69	48	Coughing for 6 days with dyspnea and irregular breathing; CT revealed hydrocephalus
P16	7d	21d	M	2 kg	7.48	26.5	9	17	130	2.4	109	44	1066	54	Low birth weight; poor responsiveness for 14 days (poor sucking strength without obvious vomiting or abdominal distension; poor mental responsiveness on the 8th day of life)

ID	Chest film	Cranial MRI	Cardiac ultrasound	Gene mutation	Ventilator-assisted ventilation	Prognosis
P42	Neonatal pneumonia	NA	NA	MMUT: c.1280G > A/ c.1677-1G > A	YES	Death
P52	bronchopneumonia	Some abnormal signal foci in white matter of bilateral parietal lobe	Patent ductus arteriosus Tricuspid regurgitation (mild-moderate)	MMUT: c.1280G > A/ c.1159A > C	YES	Discharge from hospital with improvement
P50	bronchopneumonia	Abnormal symmetry signal of basal ganglia lentiform nucleus	Patent foramen ovale	MMUT: c.323G > A/ c.1106G > A	NO	transferred to a general ward
P46	bronchopneumonia	The bilateral cerebral hemispheres had diffuse and asymmetric signal shadows in the cortex, subcortex, basal ganglia and cerebellum, white matter edema and multiple cortical neuronal necrosis	Patent foramen ovale Tricuspid regurgitation (mild-moderate)	MMUT: c.1280G > A/ c.1159A > C	YES	Withdrawal from treatment Death
P45	Bilateral lung texture enhancement	NA	Patent foramen ovale Tricuspid regurgitation (mild-moderate)	MMUT: c.682C > T/ c.1531C > T	NO	Withdrawal from treatment Sign out of hospital
P27	Bilateral bronchopneumonia, interstitial pneumonia, right pleural effusion	Hydrocephalus (severe) with periventricular interstitial leukointerstitial cerebral edema	Left ventricular myocardial and endocardial changes Regurgitation of aortic, mitral and tricuspid valves (mild)	MMACHC: c.315C > G/ c.616C > T	YES	ventriculoperitoneal-shunt Discharge from hospital with improvement
P12	Bilateral lung texture enhancement,	NA	Patent foramen ovale	MMACHC: c.658-660delAAG/ c.658-660delAAG	NO	Withdrawal from treatment Sign out of hospital
P5	Normal	Bilateral occipito-parietal cerebral hemorrhage; Bilateral putamen and thalamus have a small amount of blood infiltration;Bilateral subependymal hemorrhage	Tricuspid regurgitation	MMACHC: c.398_399delAA/ c.609G > A	YES	Withdrawal from treatment Death
P19	bronchopneumonia	Bilateral frontotemporal subdural effusion. Mild dilation of supratentorial ventricle	Patent foramen ovale	MMACHC: c.609G > A/ c.609G > A	YES	Withdrawal from treatment Death
P21	bilateral pneumonia; bilateral pleural effusion	The extracerebral space was widened, accompanied by mild expansion of supratentorial ventricles. The level of mature myelination in the brain is slightly behind that of children of the same age	Patent foramen ovale Tricuspid and pulmonary regurgitation (mild) Pulmonary hypertension (mild)	MMACHC: c.658_660delAAG/ c.658_660delAAG	YES	discharged from the hospital
P10	bronchopneumonia	Hydrocephalus (severe) accompanied by peripheral white matter interstitial cerebral edema. Multiple abnormal signals in bilateral frontal lobes, dorsal thalamus, external sac, and ventral pons	NA	MMACHC: c.217C > T/ c.658_660delAAG	NO	Withdrawal from treatment Death
P16	Bilateral lung texture enhancement	NA	NA	MMACHC: c.398_399delAA/ c.567dupT	NO	Withdrawal from treatment Death possible

The liver functions of the severe patients were almost normal. Severe acidosis was present in 3 of 7 combined MMA and in all isolated MMA patients. Pancytopenia was observed in 3 patients with isolated MMA, while the other 4 only had anemia. The C3 values of combined MMA patients ranged from 6.19 to 15.66 μmol/L, blood ammonia from 44 to 85 μmol/L, while those of isolated MMA patients ranged from 45 to 188 μmol/L, blood ammonia from 140 to 877 μmol/L, indicating higher C3 values and blood ammonia levels in isolated MMA pateints. Bronchopneumonia occurred in 4 cases of combined MMA patients and 4 cases of isolated MMA patients. Skull MR or CT scan revealed severe hydrocephalus in 2 of the 5 cases of combined MMA patients, while abnormal signals suggestive of encephalopathy were found in 3 of the 5 cases of isolated MMA patients; implying a higher risk of hydrocephalus in severe combined MMA patients.

Regarding treatment, four cases of combined MMA patients and three cases of isolated MMA patients underwent tracheal intubation and mechanical ventilation. In terms of genetic analysis, the most frequent variants among the seven cases of combined MMA were c.658-660delAAG (5/14), c.609G > A (3/14), and c.398_399delAA (2/14); all variants except for c.616C > T (1/14) were frameshift or nonsense variants. The most frequent variants among the four cases of isolated MMA patients were c.1280G > A (3/10) and c.1159A > C (2/10), and most of the variants (7/10) were missense variants, which differed from those of patients with combined MMA.

### Phenotype and genotype relationship

Among 32 patients with MMACHC gene variants (excluding those detected by newborn screening), the relationship between phenotype and genotype was not very clear. Early onset (before the age of 12 months) patients with c.609G > A homozygous or heterozygous variants were observed in 15 cases (62.5%), while late onset (after the age of 12 months) patients were observed in 6 cases (75%), which suggested that the clinical phenotypes caused by the most common variant c.609G > A were not very different. The number of cases caused by MMUT gene variants was small, so we did not compare the relationship between phenotype and genotype.

Among critically ill patients, we observed a higher occurrence of the MMACHC variant c.658-660delAAG and the MMUT variant c.1280G > A. These variants appear to be associated with a more severe phenotype. Interestingly, the MMACHC variant c.609G > A and the MMUT variant c.1159A > C were prominent hotspots across the entire cohort. However, it is essential to collect more samples to investigate this further.

## Discussion

We retrospectively analyzed the patients with MMA diagnosed in our hospital from 2014 to August 2023, mainly including combined MMA caused by MMACHC gene variants and isolated MMA caused by MMUT gene variants. 76.6% of the patients were early-onset (< 1 year of age), and therefore had more severe and typical clinical manifestations. The chief complaints of combined MMA patients were more related to the neurological system, such as psychomotor retardation, epilepsy, etc., followed by hematological system manifestations. Isolated MMA patients typically displayed poor responses, including feeding difficulties, hypotonia, and other encephalopathy symptoms. Additionally, they may experience vomiting and manifestations related to the digestive system, as well as coughing and respiratory system issues. In terms of biochemical indicators, the levels of C3 and the C3/C2 ratio were elevated in MMA patients. However, isolated MMA patients exhibited a more significant increase.. Plasma tHcy detection plays a crucial role in distinguishing between combined MMA and isolated MMA., This distinction is essential because combined MMA requires treatment with hydroxycobalamin and betaine.. Early administration of these therapies can reduce the disposal of toxic metabolites and improve neurologic complications and outcomes [15, 19]. Interestingly, studies have revealed that 58.9% of isolated MMA cases do not respond to hydroxycobalamin treatment. Additionly, this type of isolated MMA may have an earlier onset, and once clinical symptoms appear, 50% of patients experience psychomotor retardation [3]. Therefore, early intervention with special formula feeding and Levocarnitine treatment is crucial for managing this condition. Furthermore, conducting a hydroxycobalamin loading test is also necessary for isolated MMA [3].

This study identified three cases with atypical clinical manifestations. One case presented with wheezing, cyanosis, and pulmonary arterial hypertension as the initial symptoms. Previous reports have shown that combined MMA in late-onset male patients may present pulmonary arterial hypertension [20, 21]. The exact mechanism is unknown, but it may be associated with the typical pathogenic mechanisms of MMA: toxic effects of metabolic products, loss of normal metabolic products, etc. C.80A > G is a hotspot variant related to this condition. Consistent with the reports, this patient in our study was a late-onset male patient with a heterozygous c.80A > G variant. The prognosis of such patients relies on early diagnosis and treatment, including management of the primary MMA disorder [22, 23]. Therefore, we recommend that patients who are diagnosed with pulmonary arterial hypertension should receive routine metabolic screening to rule out inherited metabolic diseases. Another patient with moderate anemia had multiple visits to the hematology department, but did not undergo any metabolic tests until he lost the ability to sit independently at 11 months of age. He was finally diagnosed with MMA, a rare inherited metabolic disorder. Early diagnosis and treatment are essential for the outcome of MMA patients. Therefore, inherited metabolic diseases should be suspected as a possible cause of anemia in children who have this symptom, and relevant tests should be done promptly. A third case of MMA presented with limb weakness, and was later diagnosed with isolated MMA complicated by inflammatory demyelinating polyradiculoneuropathy. The patient is currently receiving further treatment and observation. No similar cases have been reported so far, and the exact mechanism remains unclear.

Some of the hotspot variants of MMACHC gene vary among different regions and ethnic groups. We reviewed the literature and found that in Canada representing North America and Portugal and Italy representing Europe, the hotspot variants were c.271dupA, c.394C > T and c.331C > T. While in northern, eastern and central China, the hotspot variants were c.609G > A, c.658_660delA, with only differences in the third common variant. The hotspot variants in our southern region were still c.609G > A, c.658_660delA, which were consistent with the overall mutation pattern of Chinese population. The hotspot variants of MMUT gene differed more significantly. The hotspot variants in Switzerland representing Europe were p. Asn219Tyr, while in Canada even different ethnic groups had different hotspot variants. In eastern, northern and central China, the most common variant was c.729_730insTT, with slight differences in the second common variant. While in our southern region, the hotspot variants were c.1159A > C, c.1280G > A, c.1677-1G > A, etc., which may be related to ethnicity and sample size.

We also encountered two children who were clinically and biochemically diagnosed with MMA, but no abnormalities were found in MMACHC and MMUT genes by Sanger sequencing. Through second-generation sequencing, we detected one heterozygous variant in the MMAA gene in each case, but we could not exclude the possibility of another variant in the deep intron or regulatory region in the MMAA gene. The MMAA gene encodes a protein that is involved in the intracellular processing and transport of vitamin B12 [7]. MMA caused by MMAA gene variants mainly manifests as vomiting, dyspnea, drowsiness, and most patients are sensitive to hydroxycobalamin treatment, but it cannot prevent the occurrence of neurodevelopmental disorders and chronic kidney disease [24–26]. The two children in this study also showed psychomotor retardation, vomiting, limb tremor, etc., and were sensitive to hydroxycobalamin treatment. We will continue to monitor their psychomotor development and renal changes. The c.730G > T and c.593_596delCTAG variants in the MMAA gene have not been previously reported. According to ACMG, c.730G > T variant is a variant of uncertain significance, and c.593_596delCTAG variant is a likely pathogenic variant. Both variants need further functional verification and research.

We identified one case of mitochondrial DNA depletion syndrome with mild isolated MMA due to a homozygous c.851G > A variant in the SUCLA2 gene, which is a rare condition with only 41 mutation types reported to date. The c.851G > A variant was first found in the Chinese population [27]. The SUCLA2 gene encodes a subunit of succinyl-CoA synthetase, which participates in the tricarboxylic acid cycle and mitochondrial DNA replication [8]. Therefore, variants in SUCLA2 can lead to mitochondrial DNA depletion syndrome with encephalomyopathic features and mild isolated MMA. Although we did not measure the respiratory chain (RC) enzyme activity in muscle, the patient’s clinical manifestations were in line with mitochondrial DNA depletion syndrome with mild isolated MMA [8, 28].

This study examined the biochemical, imaging, prognosis, and genetic results of critically ill patients with combined MMA or isolated MMA. Previous reports have indicated that metabolic disorders and respiratory failure are the most common causes of genetic death in the first two weeks after birth, and that methylmalonic acidemia caused by MMUT gene mutation is a major contributor to this outcome [29]. This implies that we should be vigilant to rule out methylmalonic acidemia in clinical practice when we encounter severe patients with evident acidosis, hyperammonemia, or pronounced hydrocephalus, and that we should promptly perform metabolic screening and provide symptomatic treatment.

So far, 146 types of MMACHC gene variants have been reported in the HGMD database, and no new variant types were found in this study. MMACHC variants are mostly nonsense and frameshift variants, which are related to severe clinical phenotypes. It was reported that the MMACHC c.609G > A homozygous mutation was related to early-onset [30], however in our cohort there was no difference in mutation frequency between early-onset and late-onsetdisease. Maybe this is due to limited ethnicity and sample size. In our cohort MMUT variant c.1280G > A was related to a much more severe phenotype, which was consistent with other reports [11]. MMACHC variant c.658-660delAAG also appeared to have more severe clinical phenotype, it was reported that half of the patients carrying the MMACHC variant c.658-660delAAG detected by newborn screening had poor outcome [30], which suggested that this site may have stronger pathogenicity.

In summary, we retrospectively analyzed the clinical and genetic features of patients with MMA caused by variants in the genes MMACHC, MMUT, MMAA and SUCLA2 in our center from 2014 to August 2023. We also reported a case of combined MMA with pulmonary arterial hypertension as the primary manifestation and a case of isolated MMA with limb weakness as the primary manifestation but complicated by inflammatory demyelinating polyradiculoneuropathy, suggesting that MMA may have diverse clinical manifestations involving multiple systems. We also described in detail the clinical presentations, biochemical results and genotype of 12 critically ill children, which could facilitate the diagnosis, differentiation and treatment of severe MMA cases.

**Table:** 

Wang et al. 2021 [11]
Hu et al. 2022 [5]
Lerner-Ellis et al. 2009 [12]
Nogueira et al. 2008 [13]
Liu et al. 2010 [14]
Ling et al. 2022 [15]
This study
Yu et al. 2021 [3]
Forny et al. 2016 [10]
Wang et al. 2021 [11]
Hu et al. 2022 [5]
Han et al. 2015 [16]
Skamoto et al. 2007 [17]
Worgan et al. 2006 [18]
Worgan et al. 2006 [18]
This study

## Data Availability

The datasets used in this study are not publicly available due to privacy concerns. However, interested individuals can request access to the data from the corresponding author, subject to approval by Guangzhou Women and Children’s Medical Center, Guangzhou University.

## References

[1] Fowler B, Leonard JV, Baumgartner MR (2008). Causes of and diagnostic approach to methylmalonic acidurias. J Inherit Metab Dis.

[2] Chapman KA, Gramer G, Viall S, Summar ML (2018). Incidence of maple syrup urine disease, propionic acidemia, and methylmalonic aciduria from newborn screening data. Mol Genet Metab Rep.

[3] Yu Y (2021). Different mutations in the MMUT gene are associated with the effect of vitamin B12 in a cohort of 266 Chinese patients with mut-type methylmalonic acidemia: a retrospective study. Mol Genet Genomic Med.

[4] Forny P (2021). Guidelines for the diagnosis and management of methylmalonic acidaemia and propionic acidaemia: first revision. J Inherit Metab Dis.

[5] Hu S, Kong X (2022). The genotype analysis and prenatal genetic diagnosis among 244 pedigrees with methylmalonic aciduria in China. Taiwan J Obstet Gynecol.

[6] Wang SJ, Yan CZ, Wen B, Zhao YY (2019). Clinical feature and outcome of late-onset cobalamin C disease patients with neuropsychiatric presentations: a Chinese case series. Neuropsychiatr Dis Treat.

[7] Takahashi-Iniguez T, Gonzalez-Noriega A, Michalak C, Flores ME (2017). Human MMAA induces the release of inactive cofactor and restores methylmalonyl-CoA mutase activity through their complex formation. Biochimie.

[8] Carrozzo R (2016). Succinate-CoA ligase deficiency due to mutations in SUCLA2 and SUCLG1: phenotype and genotype correlations in 71 patients. J Inherit Metab Dis.

[9] Kang L (2020). A study on a cohort of 301 Chinese patients with isolated methylmalonic acidemia. J Inherit Metab Dis.

[10] Forny P (2016). Molecular genetic characterization of 151 mut-type methylmalonic aciduria patients and identification of 41 novel mutations in MUT. Hum Mutat.

[11] Wang C (2021). Phenotypic and genotypic analysis of children with methylmalonic academia: a single-center study in China and a recent literature review. Clin Chim Acta.

[12] Lerner-Ellis JP (2009). Spectrum of mutations in MMACHC, allelic expression, and evidence for genotype-phenotype correlations. Hum Mutat.

[13] Nogueira C (2008). Spectrum of MMACHC mutations in Italian and Portuguese patients with combined methylmalonic aciduria and homocystinuria, cblC type. Mol Genet Metab.

[14] Liu MY (2010). Mutation spectrum of MMACHC in Chinese patients with combined methylmalonic aciduria and homocystinuria. J Hum Genet.

[15] Ling S (2022). The follow-up of Chinese patients in cblC type methylmalonic acidemia identified through expanded newborn screening. Front Genet.

[16] Han LS (2015). Clinical features and MUT gene mutation spectrum in Chinese patients with isolated methylmalonic acidemia: identification of ten novel allelic variants. World J Pediatr.

[17] Sakamoto O (2007). Mutation and haplotype analyses of the MUT gene in Japanese patients with methylmalonic acidemia. J Hum Genet.

[18] Worgan LC (2006). Spectrum of mutations in mut methylmalonic acidemia and identification of a common Hispanic mutation and haplotype. Hum Mutat.

[19] Fraser JL, Venditti CP (2016). Methylmalonic and propionic acidemias: clinical management update. Curr Opin Pediatr.

[20] Liu XQ (2017). Pulmonary arterial hypertension as leading manifestation of methylmalonic aciduria: clinical characteristics and gene testing in 15 cases. Beijing Da Xue Xue Bao Yi Xue Ban.

[21] Liu J (2017). Combined methylmalonic acidemia and homocysteinemia presenting predominantly with late-onset diffuse lung disease: a case series of four patients. Orphanet J Rare Dis.

[22] Chioukh FZ, Monastiri K (2019). Primary pulmonary hypertension associated with asymptomatic methylmalonic aciduria in a child. J Coll Physicians Surg Pak.

[23] Gupta A, Kabra M, Gupta N (2021). Combined methylmalonic aciduria and homocystinuria presenting as pulmonary hypertension. Indian J Pediatr.

[24] Wesol-Kucharska D (2020). Clinical picture and treatment effects in 5 patients with Methylmalonic aciduria related to MMAA mutations. Mol Genet Metab Rep.

[25] E, H. (2020). Analysis of 12 cases with methylmalonicacidemia cblA type. Zhonghua Yi Xue Yi Chuan Xue Za Zhi.

[26] Marelli C (2022). Very long-term outcomes in 23 patients with cblA type methylmalonic acidemia. J Inherit Metab Dis.

[27] Dai LF (2019). Phenotype and genotype of twelve Chinese children with mitochondrial DNA depletion syndromes. Zhonghua Er Ke Za Zhi.

[28] Stenton SL (2022). Leigh syndrome: a study of 209 patients at the Beijing Children’s Hospital. Ann Neurol.

[29] Yang L (2020). Genetic aetiology of early infant deaths in a neonatal intensive care unit. J Med Genet.

[30] He R (2020). Variable phenotypes and outcomes associated with the MMACHC c.609G>A homologous mutation: long term follow-up in a large cohort of cases. Orphanet J Rare Dis.

